# Candidiasis mucocutánea crónica, infecciones micobacterianas y rosácea en un adulto mexicano con aumento de la función en STAT1

**DOI:** 10.7705/biomedica.7521

**Published:** 2024-12-23

**Authors:** Valeria Valerio-Gómez, Uriel Pérez-Blanco, Guillermo Velázquez-Sámano, Andrea Aída Velasco-Medina, Antonio Albarrán, Itzel Yoselín Sánchez, Laura Berrón-Ruiz, Sara Espinosa-Padilla, Lizbeth Blancas-Galicia

**Affiliations:** 1 Servicio de Alergia e Inmunología, Hospital General de México “Dr. Eduardo Liceaga”, Ciudad de México, México Hospital General de México Hospital General de México Ciudad de México México; 2 Laboratorio de Inmunodeficiencias, Instituto Nacional de Pediatría, Ciudad de México, México Instituto Nacional de Pediatría Instituto Nacional de Pediatría Ciudad de México México

**Keywords:** enfermedades de inmunodeficiencia primaria, factor de transcripción STAT1, mutación con aumento de la función, candidiasis mucocutánea crónica, rosácea, tuberculosis, *Mycobacterium bovis*., primary immunodeficiency diseases, STAT1 transcription factor, gain of function mutation, candidiasis, chronic mucocutaneous, rosacea, tuberculosis, *Mycobacterium bovis*.

## Abstract

El STAT1 (*Signal Transducer and Activator of Transcription 1*) es un factor de transcripción citoplasmático, cuya función es la regulación del crecimiento, la diferenciación, la proliferación, el metabolismo y la apoptosis celular. La vía de señalización JAK/STAT, mediada por el interferon, participa en la eliminación de agentes patógenos intracelulares y virus.

Las variantes patógenas de STAT1 pueden producir una función deficiente o incrementada. El aumento de la función o actividad del factor de transcripción STAT1, descrito en el 2011, ocurre por su fosforilación excesiva. Los portadores de estas variantes patogénicas pueden desarrollar enfermedades autoinmunitarias e inflamatorias, y son susceptibles a infecciones por hongos, virus y bacterias. La manifestación temprana y común es la candidiasis mucocutánea crónica.

Este reporte trata sobre un paciente, en cuyo primer año de vida, se manifestó un aumento de la función del factor STAT1. Actualmente, tiene 27 años, ha presentado infección por el bacilo de Calmette-Guérin y *Mycobacterium tuberculosis*, candidiasis mucocutánea crónica, tiña de la cabeza (*tinea capitis*), y rosácea facial y ocular. Se descartó infección por HIV.

Por las manifestaciones clínicas, se sospechó un error innato de la inmunidad, específicamente, con aumento de la función o actividad del STAT1. El diagnóstico se corroboró con la secuenciación de múltiples genes asociados con errores innatos de la inmunidad. En este paciente, se halló la variante patogénica c.961 A>G (p.Arg321Gly) en el gen *STAT1*, previamente reportada como una mutación de aumento de la función. Finalmente, este caso ilustra que las mutaciones en los genes asociados con la inmunidad pueden contribuir a que se presenten infecciones graves y recurrentes, incluso en pacientes adultos. La candidiasis mucocutánea crónica debe hacer sospechar un aumento de la actividad del factor STAT1.

## Abstract (en)

STAT1 is a cytoplasmic transcription factor associated with cell growth regulation, differentiation, proliferation, metabolism, and apoptosis. IFN-mediated JAK/STAT signaling pathway is involved in eliminating intracellular pathogens and viruses.

However, pathogenic variants in STAT1 can result in impaired or increased function. Increased activity or function in STAT1 was described in 2011 and is characterized by excessive phosphorylation of the transcription factor. Carriers can develop autoimmune and inflammatory diseases and are susceptible to fungal, viral, and bacterial infections. The early and common manifestation is chronic mucocutaneous candidiasis.

Here, we report a clinical case of a patient with increased STAT1 activity or gain of function, which started in the first year of his life. He is currently 27 years old and has presented bacillus Calmette-Guérin and *Mycobacterium tuberculosis* infection, chronic mucocutaneous candidiasis, tinea capitis, and facial and ocular rosacea. HIV infection was ruled out.

Given the clinical manifestations, an inborn error of immunity was suspected, specifically STAT1 with gain of function. The diagnosis was corroborated by the sequencing of multiple genes associated with inborn errors of immunity. The pathogenic variant c.961 A>G (p.Arg321Gly) in the *STAT1* gene, previously reported as a gain of function mutation, was found in the patient.

Finally, this case illustrates that mutations in immune-associated genes can contribute to producing severe and recurrent infections, even in adult patients. Chronic mucocutaneous candidiasis should raise suspicion of gain of function in STAT1

El STAT1 (*Signal Transducer and Activator of Transcription* 1) es un factor de transcripción citoplasmático, que pertenece a la familia STAT y cuya función es regular el crecimiento, la diferenciación, la proliferación, el metabolismo y la apoptosis celulares ^1^. La activación del STAT1 regula la señalización celular en respuesta a múltiples ligandos, incluyendo interferon (IFN) de tipo I, II y III ^2^.

Cuando los ligandos se unen con sus receptores (INFAR1/IFNAR2), activan las cinasas Janus 1 y 2 (JAK1/JAK2), y TYK2, e inducen la fosforilación del STAT1 (que pasa a pSTATI). Este último forma un homodímero que constituye el factor de activación del IFN-y: el GAF (*Gamma-interferon Activation Factor*). Además, la unión de una molécula de pSTATI y otra de pSTAT2 forma el heterodímero denominado factor genético 3 estimulado por interferon, el ISGF3 (*Interferon-stimulated Gene Factor 3)*. El IFN de tipo I estimula la activación del ISGF3, mientras que el IFN de tipo II estimula la activación del GAF ^3^.

Los factores GAF y ISGF3 se trasladan al núcleo para unirse a los sitios de activación de interferon o GAS (*Gamma-interferon Activated Sites*) e inducir la transcripción de genes. La señalización mediada por ISGF3 actúa en la defensa del huésped contra virus, mientras que, la mediada por GAF lo hace en la eliminación de microorganismos intracelulares como las micobacterias ^2^^,^^4^.

El análisis genético de los pacientes con infecciones ha llevado a la identificación y caracterización de cuatro tipos de errores innatos de la inmunidad inducidos por alteración del factor STAT1:

- su deficiencia completa, con herencia autosómica recesiva,
- su deficiencia parcial, con herencia autosómica recesiva,
- su deficiencia, por herencia autosómica dominante, y
- aumento de su función o actividad, de herencia autosómica dominante ^4^^,^^5^.

El aumento de la actividad del STAT 1 se describe en inglés como *gain of function*. Este resulta por una falta de desfosforilación del STAT1, que lleva a su hiperfosforilación en el núcleo y hace que los genes estimulados por el IFN se continúen transcribiendo ^2^^-^^4^. Esta condición se ha asociado con una amplia variedad de manifestaciones infecciosas y autoinmunitarias. Los pacientes son susceptibles a infecciones por hongos, bacterias y virus. La infección más temprana y frecuente es la candidiasis mucocutánea crónica ^6^. Las infecciones bacterianas incluyen las micobacterianas (*Mycobacterium tuberculosis* o bacilo de Calmette-Guérin), y las cutáneas como foliculitis o forunculosis ^7^. También se presentan infecciones por herpes simple y varicela zóster ^3^^,^^6^. Entre las manifestaciones autoinmunitarias más frecuentes, están el hipotiroidismo, la diabetes mellitus de tipo 1, la anemia hemolítica autoinmunitaria y el lupus eritematoso sistémico ^8^.

Se presenta un paciente con aumento de la actividad de STAT1 y manifestaciones infecciosas desde el primer año de vida, con diagnósticos de candidiasis mucocutánea crónica, tuberculosis pulmonar, herpes zóster y rosácea, a los 27 años.

## Consideraciones éticas

Los autores declaran que los procedimientos fueron realizados según las normas éticas, el Reglamento de la Ley General de Salud en materia de Investigación para la Salud y la Declaración de Helsinki. Los autores declaran que han seguido los protocolos de su centro de trabajo sobre la publicación de datos de pacientes y la firma del consentimiento informado.

## Caso clínico

**Figura 1.**
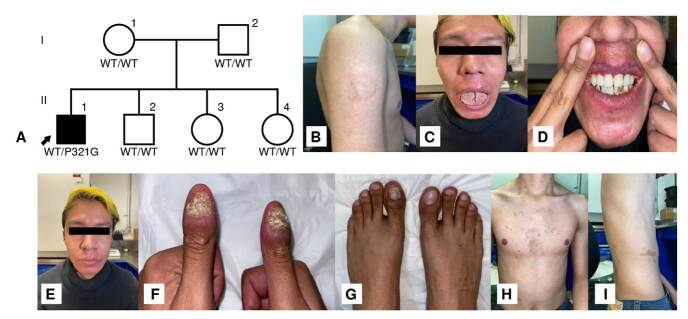
Árbol genealógico y hallazgos clínicos. **A**) Pedigrí familiar. El paciente se representa con un cuadro negro. **B**) Cicatriz hipertrófica como secuela de infección por el bacilo de Calmette-Guérin. **C**) Candidiasis pseudomembranosa en la lengua, glositis, fisuras linguales y queilitis angular. **D**) Eritema gingival linear. E) Rosácea. Se aprecia eritema peribucal faciales, acompañados de pápulas en nariz y mejillas. **F-G**) Onicomicosis candidiásica, onicolisis, distrofia ungueal y onicogrifosis con edema periungueal. H-l) Se observan zonas hiperpigmentadas en tórax y abdomen, secundarias a infección por herpes zóster.

Se trata de un hombre de 27 años, originario y residente de la Ciudad de México, con padres y tres hermanos aparentemente sanos (figura 1 A). Recibió la vacuna con el bacilo de Calmette-Guérin (BCG) al nacimiento, pero presentó una reacción adversa a los dos meses de vida, con adenitis en ambas axilas y falta de cicatrización en el sitio de aplicación de la vacuna; mejoró mediante la administración de rifampicina durante ocho meses, pero le quedó una cicatriz queloide (figura 1B).

A los nueve meses, desarrolló candidiasis oral y en el área del pañal. Aunque ambas remitieron con el tratamiento, eran recurrentes. La candidiasis oral persiste hasta hoy, y le afecta la lengua y las encías (figura 1, C y D).

A los siete años, presentó tiña en el cuero cabelludo, la cual mejoró con un tratamiento no especificado.

Desde los 10 años ha presentado rosácea en el rostro, inicialmente con pápulas y eritema en la piel de las mejillas y la nariz, y actualmente con cambios crónicos (figura 1E).

A los 15 años presentó onicomicosis, recibió múltiples tratamientos antifúngicos, sin mejoría y con persistencia hasta la fecha (figura 1 F y G). Ha presentado rosácea ocular desde la infancia y, también, padece de asma y rinitis alérgica.

Sus pruebas cutáneas fueron positivas para ácaros, epitelios de animales y polen. Se administraron esteroides tópicos y broncodilatadores inhalados.

**Figura 2.**
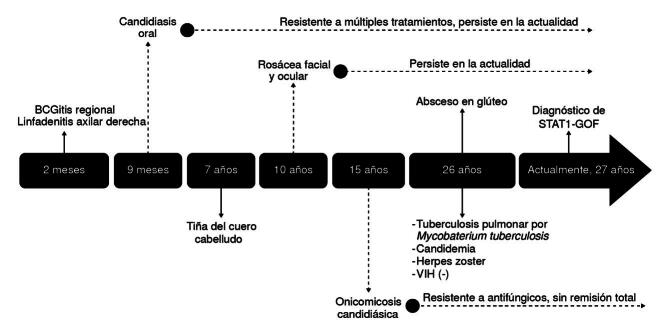
Evolución clínica del paciente. Línea de tiempo que ilustra las manifestaciones de la enfermedad, desde la primera hasta la actualidad.

Por el carácter crónico de la candidiasis, se consideró una alteración del sistema inmunológico, se descartó infección por HIV y se le informó que padecía una enfermedad granulomatosa crónica (se desconoce en qué se fundamentó el diagnóstico) (figura 2).

A los 26 años, presentó fiebre, pérdida de peso, astenia, adinamia, hiporexia, tos, hemoptisis, disnea y un absceso glúteo. En el servicio de urgencias, el paciente estaba en mal estado general, con desnutrición, palidez de piel y mucosas, dificultad respiratoria, saturación de oxígeno del 87%, rosácea en cara, lengua y mucosa oral con placas blancas, eritema en encías, cicatriz de la vacuna BCG de 5 cm de diámetro, hipoventilación pulmonar, y onicomicosis en manos y pies.

**Figura 3.**
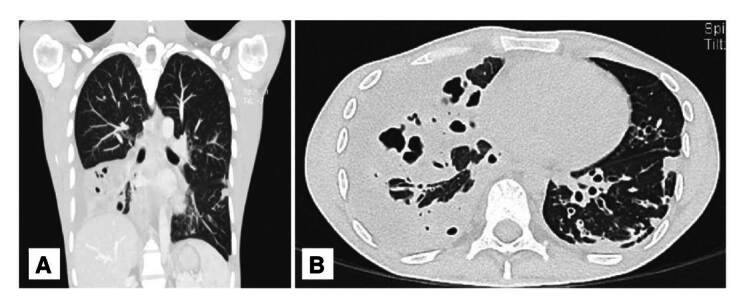
Tomografía de tórax con contraste, en corte coronal **(A)** y transversal **(B)**, en la que se aprecia consolidación del lóbulo inferior del pulmón derecho acompañada de cavitaciones pequeñas, bronquiectasias bilaterales, derrame pleural derecho y broncograma aéreo

En la tomografía de tórax, se observaron imágenes correspondientes a derrame pleural derecho y neumonía multifocal (figura 3). El ultrasonido de glúteo mostró un absceso profundo. Los resultados de laboratorio evidenciaron anemia, leucocitosis a expensas de neutrofilia y elevación de la proteína C reactiva (cuadro 1). La prueba de dihidrorrodamina fue normal, con lo que se descartó el diagnóstico previamente establecido de enfermedad granulomatosa crónica (figura suplementaria 1).

**Cuadro 1.** Hallazgos de laboratorio del paciente

Parámetro	Valor	Intervalo de referencia
Hemoglobina (g/dl)	9,7	13a 17
Hematocrito (%)	30,5	42 a 52
Leucocitos (células/μl)	26.700	4.500 a 10.000
Neutrófilos (células/μl)	23.500	2.500 a 7.000
Linfocitos (células/μl)	1.300	1.000 a 4.000
Plaquetas (células/μl)	558.000	150.000 a 400.000
Proteína C reactiva (mg/L)	204	<5
Procalcitonina (ng/ml)	0,89	0,25 a < 50

El cultivo de la muestra tomada de la cavidad oral y el hemocultivo, tuvieron crecimiento de Candida albicans; en el cultivo de esputo, se obtuvo C*. albicans y C.krusei*, el cultivo de líquido broncoalveolar fue negativo.

Se inició tratamiento con vancomicina para el absceso, con el cual presentó mejoría. El paciente recibió ceftriaxona, claritromicina y fluconazol, pero persistió la fiebre. Se practicó una biopsia pleural, en la cual se detectó M. tuberculosis mediante PCR. Se inició la fase intensiva del esquema antituberculoso, o antifímico, con isoniacida, rifampicina, pirazinamida y etambutol, obteniéndose una adecuada mejoría. Durante esta hospitalización, también desarrolló herpes zóster que remitió con 10 días de valaciclovir (figura 1, H y I). Actualmente, el paciente se encuentra completando su tratamiento antituberculoso.

**Figura 4.**
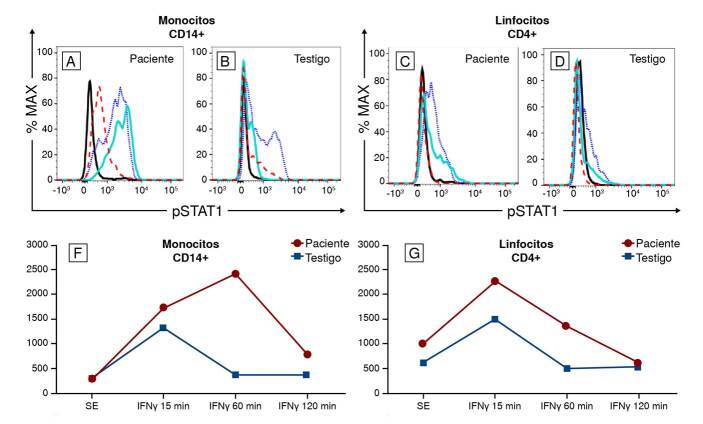
Análisis de la fosforilación del STAT1 (pSTATI) por citometría de flujo. Histogramas que muestran la intensidad media de fluorescencia del pSTATI en monocitos CD14+ (A, B) y linfocitos T CD4+ (C, D) del paciente (A, C) y el testigo (B, D). La línea continua negra muestra las células sin estímulo y, después de estimularlas con IFN-y durante 15 minutos (línea punteada azul), durante 60 minutos (línea continua turquesa) y durante120 minutos (línea discontinua roja). Se observa retraso en la desfosforilación del STAT1 en el paciente, en comparación con la del testigo. Se muestra la intensidad media de fluorescencia del pSTATI en monocitos CD14+ (F) y linfocitos T CD4+ (G), del paciente y el testigo, tras la estimulación con IFN-y a los 15, 60 y 120 minutos. Se observa hiperfosforilación del STAT1 en el paciente, en comparación con el testigo.

Revaluando el caso, por los antecedentes de infección por el bacilo de Calmette-Guérin, tuberculosis, herpes zóster, candidiasis mucocutánea y rosácea, el diagnóstico se orientó hacia un aumento de la función del STAT1 (figura 4). Se solicitó un panel de genes de errores innatos de la inmunidad, por secuenciación de nueva generación, y se halló la variante patogénica c.961A>G (p.Arg321Gly) en el gen *STAT1*, asociada con aumento de la función del STAT1. Sus padres y tres hermanos no portan la mutación (WTV WT) gen STAT1.

## Discusión

En la literatura científica revisada, se han reportado 12 casos en México con aumento de la función del STAT1 (cuadro 2) ^9^^-^^13^. En el presente informe, se identificó una variante patogénica de novo en el gen *STAT1*, con aumento de la función, en un paciente con candidiasis mucocutánea crónica, infección por bacilo de Calmette-Guérin, tuberculosis pulmonar, herpes zóster y rosácea. La variante patogénica c.961A>G (p.Arg321GIy) en el STAT1 ya fue descrita en tres pacientes de una cohorte internacional de 274 casos. En ellos, la candidiasis mucocutánea crónica fue la manifestación más temprana (a los dos, tres y seis meses de vida, respectivamente) ^6^ al igual que en el caso aquí reportado (nueve meses).

Los interferones de tipo I y II inhiben el desarrollo de las células Th17 (productoras de IL-17) ^3^^,^^14^. Los pacientes con aumento de la función del STAT1 tienen incrementada la reacción al IFN de tipo I y II, por lo que tienen pocas células Th17. La IL-17 es una citocina clave en la inmunidad contra hongos, lo cual podría explicar la predisposición a infecciones fúngicas crónicas y recurrentes, principalmente por Candida spp., en la mucosa oral y genital, las uñas y la piel ^8^^,^^14^. La penetrancia genética es completa en los pacientes con aumento de la función del *STAT1*. En una cohorte de 274 pacientes, el 98 % desarrolló candidiasis mucocutánea crónica en el primer año de vida y el 2 % restante presentó otras manifestaciones diferentes a dicha infección ^6^.

Los tres pacientes reportados con la variante c.961A>G (p.Arg321 Gly) por Toubiana *et al*. ^6^, no presentaron infecciones micobacterianas, mientras que el reportado aquí exhibió infección local y regional por el bacilo de Calmette-Guérin e infección pulmonar por *M. tuberculosis*. El mecanismo inmunológico que explica la susceptibilidad a infecciones micobacterianas permanece incierto. Sin embargo, se conoce que el IFN-y es una citocina crítica para la eliminación de agentes patógenos intramacrofágicos, como *M. tuberculosis* u otro tipo de micobacterias. Liu *et al*. observaron un porcentaje reducido de células productoras de IFN-y en pacientes con aumento de la función del STAT1, en comparación con los controles sanos ^14^.

Las infecciones virales se presentan en un tercio de los pacientes con aumento de la función del STAT1. En la cohorte de 274 pacientes, al menos el 38 % desarrolló una infección viral sistémica o infecciones virales mucocutáneas recurrentes ^3^^,^^6^. Las principales causas de las mucocutáneas fueron el virus de la varicela zóster y el del herpes simple, mientras que la infección viral sistémica se asoció principalmente con citomegalovirus o virus de Epstein-Barr ^6^. Durante su hospitalización por tuberculosis pulmonar y absceso glúteo, el paciente reportado aquí presentó herpes zóster, el cual se resolvió con tratamiento antiviral.

En el espectro clínico del aumento de la función del STAT1, se incluyen las enfermedades autoinmunitarias. Estas se han reportado en el 43 % de los pacientes, con una edad media de 24 años. Entre las manifestaciones clínicas están las de hipotiroidismo (la más frecuente), hipertiroidismo, diabetes mellitus de tipo 1, lupus eritematoso sistémico, anemia hemolítica autoinmunitaria, trombocitopenia, enfermedad de Crohn, colitis ulcerativa, vitíligo y alopecia, entre otras ^3^^,^^6^^,^^8^. El paciente de este reporte aún no ha presentado fenómenos autoinmunitarios; sin embargo, se le debe hacer seguimiento para detectar indicios clínicos y paraclínicos sugestivos.

Los pacientes con aumento de la función del STAT1 pueden presentar infecciones dermatológicas por dermatofitos como *Trichophyton* spp. y *Microsporum* spp. ^6^. Otras complicaciones en la piel incluyen foliculitis, celulitis, abscesos y paroniquia, con *Staphylococcus aureus* como el principal agente bacteriano ^6^. El caso aquí reportado presentó *tinea capitis* y un absceso profundo en el glúteo.

La rosácea es una enfermedad inflamatoria crónica de la piel, multicausal, que afecta las convexidades del rostro, con posibilidad de afectación ocular ^15^. Los pacientes con aumento de la función del STAT1 presentan inicialmente rosácea en la cara y en los ojos desde la infancia, pero son mal diagnosticados dermatológica y ocularmente. Estos pacientes tienen una mayor proliferación de *Demodex* spp., pero se desconoce el mecanismo por el cual se produce la rosácea ^13^. La rosácea en asociación con las infecciones, hicieron sospechar el diagnóstico clínico, el cual fuá corroborado genéticamente.

En aquellos pacientes con infecciones crónicas y recurrentes, se debe descartar un defecto de la inmunidad, ya sea secundario o congénito. Además de las del STAT1, se han documentado mutaciones en otros genes responsables de errores innatos de la inmunidad y que predisponen a candidiasis mucocutánea crónica, como *STAT3, AIRE, CLEC7A, CARD9, RORC, ACT1, IL-17RA, IL-17RC,* e *IL-17F*^16^.

Finalmente, el tratamiento es crucial para mejorar el pronóstico y disminuir la mortalidad. El tratamiento se basa en medidas terapéuticas de soporte, como profilaxis antifúngica a largo plazo (local o sistémica) administrada en aproximadamente el 74 % de los pacientes. El fluconazol es el principal agente antifúngico, seguido por el itraconazol y el posaconazol ^6^. Sin embargo, se ha reportado que, en hasta el 39 % de los pacientes, se presenta resistencia contra, al menos, un agente antifúngico, por lo cual se requieren opciones de segunda línea, como voriconazol, terbinafina, caspofungina o anfotericina B ^6^. El paciente del presente caso no recibió un tratamiento profiláctico antifúngico debido a la falta de un diagnóstico definitivo.

Actualmente, se ha estudiado la eficacia de los inhibidores de JAK, como ruxolitinib o baricitinib, en pacientes con aumento de la función del STAT1 que cursa con manifestaciones infecciosas, inflamatorias y autoinmunitarias graves. Deyá-Martínez *et al*. ^17^ reportaron una cohorte retrospectiva de 10 pacientes pediátricos (todos con candidiasis mucocutánea crónica y al menos una manifestación autoinmunitaria o inflamatoria) con diagnóstico genético de aumento de la función del STAT1 y tratamiento con inhibidores de JAK, en la que el 90 % (n = 9) mostró respuestas terapéuticas parciales o completas; solo un paciente no completó el tratamiento por presentar eventos adversos. Existen otros estudios que aportan evidencia suficiente de la eficacia de los inhibidores de JAK en el tratamiento de la candidiasis mucocutánea crónica y la disminución de las manifestaciones autoinmunitarias o inflamatorias ^18^^,^^19^. El paciente aquí reportado no ha recibido inhibidores de JAK, pero podrían considerarse en un futuro.

El trasplante de células progenitoras hematopoyéticas es una opción de tratamiento curativo en pacientes con síntomas graves. Sin embargo, los eventos adversos graves secundarios a la falla del injerto, hacen que el pronóstico empeore o que no se recomiende en diferentes errores innatos de la inmunidad, incluyendo el aumento de la función del STAT1.

En una revisión sistemática de 442 pacientes con esta condición, 25 fueron sometidos a trasplante de progenitores hematopoyéticos. De estos, 11 (44 %) fallecieron meses después del trasplante y los pacientes en los que el trasplante fue exitoso tuvieron remisión de la enfermedad ^8^. Sin embargo, el 60 % de aquellos con injerto exitoso, fallecieron tres años después del trasplante ^8^. Estos hallazgos sugieren que el trasplante de progenitores hematopoyéticos es una opción curativa potencial del aumento de la función del STAT1, pero se asocia con riesgo de falla del injerto y muerte. Por ende, actualmente no se considera una opción terapéutica de primera línea para dichos pacientes; podría considerarse solo en pacientes con síntomas graves y persistentes. El paciente de este reporte no ha sido considerado para recibir trasplante.

Los pacientes con errores innatos de la inmunidad son más susceptibles a infecciones, manifestaciones autoinflamatorias y autoinmunitarias. El aumento de la función del STAT1 es un ejemplo de un error innato en el que hay incremento de la actividad de una molécula, en lugar de una deficiencia. Las manifestaciones clínicas se inician desde el nacimiento, pero el diagnóstico suele retrasarse por décadas. Debido a que el aumento de la función del STAT1 tiene un patrón de herencia de penetrancia completa, todos los pacientes estarán afectados. Se sugiere hacer el diagnóstico diferencial de aumento de la función del STAT1 en aquellos pacientes con candidiasis mucocutánea crónica. El acceso a la secuenciación de nueva generación (secuenciación múltiple de genes responsables de errores innatos de la inmunidad), permite este tipo de diagnóstico.

## Figures and Tables

**Figura suplementaria 1.**
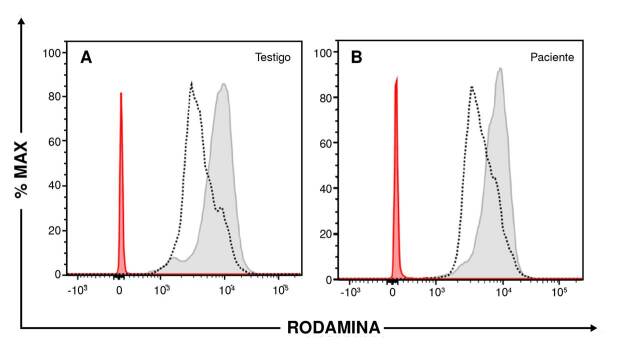
Prueba de la oxidación de la dihidrorrodamina 123 en el paciente y en un testigo. En cada una las figuras, se observan tres histogramas: el rojo sólido representa las células sin tinción o en estado basal del control (testigo, A) y del paciente (B); el gris sólido muestra la estimulación de los neutrófilos con PMA y la línea negra punteada representa la población de neutrófilos estimulada con zimosán. Ambos gráficos muestran la oxidación de la dihidrorrodamina. Se observa un incremento similar de la intensidad media de fluorescencia en el control y el paciente.

PMA: Phorbol 12-myristate 13-acetate

## References

[1] Hu X, Li J, Fu M, Zhao X, Wang W (2021). The JAK/STAT signaling pathway: From bench to clinic. Signal Transduct Target Ther.

[2] Luo Y, Alexander M, Gadina M, O’Shea JJ, Meylan F, Schwartz DM (2021). JAK-STAT signaling in human disease: From genetic syndromes to clinical inhibition. J Allergy Clin Immunol.

[3] Okada S, Asano T, Moriya K, Boisson-Dupuis S, Kobayashi M, Casanova JL (2020). Human STAT1 gain-of-function heterozygous mutations: Chronic mucocutaneous candidiasis and type I interferonopathy. J Clin Immunol.

[4] Asano T, Utsumi T, Kagawa R, Karakawa S, Okada S (2023). Inborn errors of immunity with loss- and gain-of-function germline mutations in STAT1. Clin Exp Immunol.

[5] Bousfiha A, Moundir A, Tangye SG, Picard C, Jeddane L, Al-Herz W (2022). The 2022 Update of IUIS Phenotypical classification for human inborn errors of immunity. J Clin Immunol.

[6] Toubiana J, Okada S, Hiller J, Oleastro M, Lagos-Gómez M, Aldave-Becerra JC (2016). Heterozygous STAT1 gain-of-function mutations underlie an unexpectedly broad clinical phenotype. Blood.

[7] Depner M, Fuchs S, Raabe J, Frede N, Glocker C, Doffinger R (2016). The extended clinical phenotype of 26 patients with chronic mucocutaneous candidiasis due to gain-of-function mutations in STAT1. J Clin Immunol.

[8] Zhang W, Chen X, Gao G, Xing S, Zhou L, Tang X (2021). Clinical relevance of gain-and loss-of-function germline mutations in STAT1: A systematic review. Front Immunol.

[9] Pedraza-Sánchez S, Jl Méndez-León, González Y, Ventura-Ayala ML, Herrera MT, Lezana- Fernández JL (2018). Oral administration of human polyvalent IgG by mouthwash as an adjunctive treatment of chronic oral candidiasis. Front Immunol.

[10] Luis BAL, Calva-Mercado JJ (2018). Recurrent spontaneous intestinal perforation due to STAT1- GOF mutation. Am J Gastroenterol.

[11] Pedraza-Sánchez S, Lezana-Fernández JL, González Y, Martínez-Robles L, Ventura-Ayala ML, Sadowinski-Pine S (2017). Disseminated tuberculosis and chronic mucocutaneous candidiasis in a patient with a gain-of-function mutation in signal transduction and activator of transcription 1. Front Immunol.

[12] Staines-Boone AT, Vignesh P, Tsumura M, de la Garza-Fernández G, Tyagi R, Rawat A (2023). Fatal COVID-19 infection in two children with STAT1 gain-of-function. J Clin Immunol.

[13] Saez-de-Ocariz M, Suárez-Gutiérrez M, Migaud M, Farrill-Romanillos PO, Casanova JL, Segura-Méndez NH (2020). Rosacea as a striking feature in family members with a STAT1 gain-of-function mutation. J Eur Acad Dermatol Venereol.

[14] Liu L, Okada S, Kong XF, Kreins AY, Cypowyj S, Abhyankar A (2011). Gain-of-function human STAT1 mutations impair IL-17 immunity and underlie chronic mucocutaneous candidiasis. J Exp Med.

[15] Ahn CS, Huang WW (2018). Rosacea pathogenesis. Dermatol Clin.

[16] Ma Y, Wang X, Li R (2022). AIRE gene mutation predisposing chronic mucocutaneous candidiasis and pigmented retinitis in two kids from a Chinese family. Emerg Microbes Infect.

[17] Deyà-Martínez A, Rivière JG, Roxo-Junior P, Ramakers J, Bloomfield M, Guisado HP (2022). Impact of JAK inhibitors in pediatric patients with STAT1 gain-of-function (GOF) mutations-10 children and review of the literature. J Clin Immunol.

[18] Bloomfield M, Kanderová V, Paracková Z, Vrabcová P, Svatoñ M, Froňková E (2018). Utility of ruxolitinib in a child with chronic mucocutaneous candidiasis caused by a novel STAT1 gain-of-function mutation. J Clin Immunol.

[19] Forbes LR, Vogel TP, Cooper MA, Castro-Wagner J, Schussler E, Weinacht K (2018). Jakinibs for the treatment of immune dysregulation in patients with gain-of-function signal transducer and activator of transcription 1 (STAT1) or STAT3 mutations. J Allergy Clin Immunol.

